# Cognitive Bias Modification Training to Improve Implicit Vitality in Patients With Breast Cancer: App Design Using a Cocreation Approach

**DOI:** 10.2196/18325

**Published:** 2021-03-10

**Authors:** Roos Wolbers, Christina Bode, Ester Siemerink, Sabine Siesling, Marcel Pieterse

**Affiliations:** 1 Centre for eHealth and Wellbeing Research University of Twente Enschede Netherlands; 2 Saxion University of Applied Science Enschede Netherlands; 3 Department of Internal Medicine Ziekenhuis Groep Twente (ZGT) Hengelo Netherlands; 4 Department of Research and Development Netherlands Comprehensive Cancer Organization (IKNL) Utrecht Netherlands; 5 Department of Health Technology and Services Research Technical Medical Centre University of Twente Enschede Netherlands

**Keywords:** breast cancer, cognitive bias modification, eHealth, fatigue, oncology, psychology, vitality

## Abstract

**Background:**

More than 50% of all patients with breast cancer experience fatigue symptoms during and after their treatment course. Recent evidence has shown that fatigue is partly driven by cognitive biases such as the self-as-fatigued identity bias, which may be corrected with computer-based cognitive bias modification (CBM) techniques.

**Objective:**

The aim of this study was to design a CBM-training app by adopting a cocreation approach.

**Methods:**

Semistructured interviews were conducted with 7 health care professionals, 3 patients with breast cancer, and 2 patient advocates. The aim of the interviews was to collect input for the design of the CBM training, taking the values and preferences of the stakeholders into account, and to determine the timing and implementation of the training in the treatment course.

**Results:**

Overall, the interviews showed that the concept of CBM was accepted among all stakeholders. Important requirements were revealed such as the training needs to be simple and undemanding, yet engaging and persuasive. Based on the results, an eHealth app IVY (Implicit VitalitY) was created. The findings from the interviews suggested that IVY should be offered early in the breast cancer treatment course and should be carefully aligned with clinical treatment.

**Conclusions:**

The findings of this study show that using CBM as a preventive approach to target cancer-related fatigue is an innovative technique, and this approach was embraced by breast cancer stakeholders. Our study suggests that CBM training has several benefits such as being easy to use and potentially increasing perceived self-control in patients.

## Introduction

Cancer-related fatigue is a highly prevalent and pervasive symptom that has a major impact on the quality of life [1]. More than 50% of all patients with breast cancer experience fatigue complaints during their treatment course, with a substantial proportion retaining serious fatigue symptoms after treatment [2]. Patients report fatigue as more distressing than other cancer-related symptoms such as nausea and pain [1,3]. Furthermore, fatigue is directly related to limitations in occupational participation [4], social participation, and even in performing the daily routine [5].

Cancer-related fatigue is a complex symptom, in which the etiology is multifactorial and not entirely clarified. It is characterized as “an unusual, persistent, subjective sense of tiredness related to cancer or cancer treatment that interferes with usual functioning” [3]. Daily limitations can be related to physical functioning, mood, motivation, cognitions, and social functioning, including interindividual variations. The start, development, and course of cancer-related fatigue are rarely studied. Regarding fully developed serious fatigue symptoms, one of the underlying mechanisms seems to be the psychological distortion in perception, also known as cognitive biases. Previous research studies have suggested that breast-cancer survivors have an attentional bias for cancer-related words compared to healthy individuals [6]. In a similar way, patients with breast cancer can develop a distorted fatigue self-concept (“I am a person with low energy”). This may implicitly consolidate and aggravate fatigue-related behavior and consequently, fatigue as a symptom. These cognitive biases are largely implicit and occur outside awareness. The adverse effect of cognitive biases can be both direct (due to the higher sensitivity to signals of fatigue) and indirect (fatigue-related behavior is unconsciously avoided, resulting in self-imposed limitations and a decrease in physiological capacity) as has been found for pain by Crombez et al [7] and for fatigue by Hughes and colleagues [8]. In order to prevent the full development of serious fatigue symptoms and related behavior, interventions are needed that can be applied early in the process of development of cancer-related fatigue and accompanying cognitive biases. The existing interventions for cancer-related fatigue are reactive and focus on managing existing fatigue symptoms. Our study, in contrast, focuses not only on reducing the existing fatigue but also on pre-emptively enhancing vitality in patients, to make them more resilient during treatment against developing fatigue.

An emerging technique to directly correct or prevent maladaptive biases with simple repetitive association tasks is cognitive bias modification (CBM). CBM programs are aimed at retraining learnt thought patterns (biases). The underlying theories (dual process models) describe human behavior, thoughts, and feelings as the integration of conscious reflexive processes on the one hand and automated and unconscious processes on the other hand [9]. Existing interventions aimed at self-management or rehabilitation primarily target reflexive processes in patients. The method proposed here (CBM) targets automated unconscious processes and can therefore be considered as complementary to current fatigue treatments.

CBM has already shown beneficial effects in reducing anxiety, depression, chronic pain, and addiction [10-12]. The most robust evidence for cognitive bias in the context of fatigue is *attentional bias*. In a systematic review, Hughes et al [8] concluded that patients with chronic fatigue syndrome consistently show an attentional bias toward health-threatening cues compared to healthy controls. This is supported by evidence from research on insomnia [13], a condition closely related to fatigue. While attentional bias represents a perceptual orientation toward fatigue cues, s*elf-identity bias* reflects a more elaborate interpretative cognitive process*.* As has been shown in pain research, automatically activated associations between pain and “self” play a role in the perception of pain severity [14]. Self-identity is conceptually closely related to a self-schema [15], defined as a person’s integrated set of memories, beliefs, and generalizations about his behavior in a certain domain. When patients experience fatigue symptoms over an extended period, either during or following their treatment, they are at risk of developing a schema of the self as a “tired person.” Based on evidence from various domains [16,17], it is evident that such self-schemas are strongly rooted within the implicit system and should therefore also be treated at the implicit level. CBM interventions do exactly this. We explore the assumed treatment mechanism (self-identity bias modification to improve fatigue in patients with breast cancer) and work on a theoretical substantiation of the working mechanism for fatigue in this patient group. A theoretical model that informs our conceptual work is the Schema Enmeshment Model of pain proposed by Pincus and Morley [17]. This theory describes the onset and mechanisms of information-processing biases as the intersection of 3 psychological schemas representing the symptom, the illness, and the self. Repeated simultaneous activation of these schemas is hypothesized to result in gradual enmeshment of these schemas, where activation of an element within one schema automatically spreads to the other schemas. Assuming that the Pincus Schema Enmeshment model is equally applicable to the psychosomatic symptom of fatigue, the self-as-fatigued identity bias was chosen as the target in this study.

Based on the hypothesized relationships and working mechanisms, cognitive biases seem to play a role in developing and maintaining fatigue. Therefore, it seems warranted to study whether CBM can be beneficial in reducing and preventing cancer-related fatigue. Furthermore, it might strengthen vitality-related cognitive bias to further buffer the fatigue-stimulating processes. Therefore, the aim of this project was to develop a CBM-training app to increase vitality bias and reduce fatigue bias in patients with breast cancer. The focus of this training will be on retraining fatigue and vitality self-concept associations by letting patients repeatedly pair vitality-related words with “self” and fatigue-related words with “others.” The words that we used for CBM were general words describing fatigue and vitality. We explicitly do not use words that are linked to only 1 dimension of fatigue in order to maximize the ability for identification. Offering this training at an early stage of treatment or even pre-emptively by strengthening a vitality-rich self-concept, thereby creating a “buffer” against fatigue symptoms could help making patients more resilient during and after treatment.

Although CBM is a promising technique, acceptance by patients and health care professionals has not yet been systematically studied. Beard et al [18] studied attitudes toward CBM in patients with anxiety disorders. The perceived advantages of CBM were that the tasks were easy and straightforward. However, the barriers to CBM were that the tasks were repetitive and boring. Furthermore, patients lacked understanding about the purpose of the tasks and thought it was strange and not credible. In particular, in the domain of somatic diseases, psychosocial treatments offered to alleviate psychosomatic symptoms such as fatigue are known to evoke feelings of self-blame in patients [19]. Taking into account the possible resistance in stakeholders with regard to CBM, it seems essential to involve patients and health care professionals throughout the design process [20]. This cocreation approach, based on user-centered design principles, has been shown to lead to more acceptance of systems, better adherence, greater user satisfaction, and better implementation chances of the technology [21].

In this cocreation process, it is first important to obtain insight into the perspective of patients with breast cancer and health care professionals on fatigue and how they perceive the course of fatigue, to determine at which stage of the patient’s journey a preventive CBM-vitality training can best be offered to patients. One possibility is that the training should be offered as early in the treatment course as possible in order to obtain the best preventive results. However, in this early phase, patients have just been diagnosed and are preparing for their first treatment, which makes it less likely that patients will be open to use an intervention. Furthermore, the motivation to receive training will probably be higher when the patients experience the first fatigue signals. Therefore, the first aim of this study was to explore the window of opportunity for introducing the CBM intervention in the early stage. The second aim of this study was to examine the attitude of the stakeholders with regard to the concept of CBM to understand the main drivers and barriers for accepting this technique. The third aim was to gather information about their needs and values to formulate concrete design requirements. Finally, it was important to gain more insight into how the CBM training can be successfully implemented in regular clinical care. This leads to the following 4 research questions:

1. What is the preferred moment in the patient’s journey to introduce the CBM treatment pre-emptively?

2. What is the attitude among patients and health care professionals toward the concept of CBM aimed at preventing chronic fatigue in patients?

3. What are the requirements that need to be taken into account in the design of the CBM training?

4. How can the training be fitted into the regular clinical treatment regimen in order to facilitate sustainable implementation?

## Methods

### Participants in This Study

Twelve participants were recruited by purposive sampling [22]. Participants were patients with breast cancer (n=3), patient advocates from BVN (Dutch breast cancer patient association) (n=2), and health care professionals involved in the treatment of patients with breast cancer (n=7). Patient advocates were patients with breast cancer too, but they were invited as representatives and stakeholders of BVN. These patient advocates were following an education program and had insight into the patients’ needs and wishes from not only their own situations and experiences but also from many breast cancer types and treatment forms.

Recruitment was stopped when data saturation was achieved and no new information was obtained from the interviews. Patients with breast cancer from a large regional hospital with a breast cancer unit (Ziekenhuis Groep Twente [ZGT]) were included. In 2019, 306 patients from this breast cancer unit have had breast cancer surgery. The mean age of this patient population was 61.8 (SD 13.9) years, which is comparable with the Dutch benchmark from the Dutch Institute for Clinical Auditing with a mean age of 61.6 (SD 12.9) years. Inclusion criteria were that patients were diagnosed with breast cancer and had finished or almost finished receiving cancer treatment. The first patient had finished chemotherapy and was receiving radiotherapy. The second patient was in the last phase of chemotherapy. The third patient had finished treatment several years ago. Health care professionals who participated were a medical oncologist, oncology physiotherapist, nurse practitioner, surgical oncologist, general practitioner, and 2 oncology nurses. Participants were selected by the medical oncologist and approached via telephone by the researcher RW.

### Data Collection

Semistructured face-to-face interviews were conducted by RW with patients, patient advocates, and health care professionals. The aim of the interviews was to gain more insight into the course of the fatigue, explore the attitude of the stakeholders toward CBM, collect the requirements for the training based on preferences, and lastly investigate how the training could be implemented into regular care. An overview of the interview topics and questions can be found in Table 1. Interview questions were generally the same for patients, patient advocates, and professionals and were focused on their expertise. In the interviews, the idea of CBM was introduced to the participants with an oral explanation. The literal translation of the original Dutch text is added in Multimedia Appendix 1. To help them understand the concept of CBM, the mobile approach avoidance CBM training “Breindebaas” for alcohol addiction was shown as an example [23]. Interviews lasted for 30-60 minutes and were recorded with a voice recorder. The interviews were conducted in Dutch and back-translated to English. After the interviews, the first version of the platform was developed by software engineers and shown to the same patients for feedback. Two out of 3 patients were willing to test the platform and provide feedback.

**Table 1:** Overview of the interview topics.

Interview topic	Topics of the questions^a^
Demographics and medical information	Age, occupation, diagnosis, and treatment
Fatigue	Impact and course of fatigue
Attitude	Attitude and experience supporting health by technology and opinion on cognitive bias modification training
Preferences	Preferred platform, content, layout, and explanation of the training
Implementation	Integration of training in regular care and the best moment for offering it to patients

^a^The literal translation of the main questions is given in Multimedia Appendix 1.

### Data Analysis

The audio recordings were transcribed verbatim. After transcription, thematic analysis was conducted [24]. This process started with familiarization of the data and generation of the initial codes. Then, the codes were transformed into themes and subthemes. This was done until no new codes emerged, indicating that the saturation point was reached. Interrater reliability was ensured by means of consensus estimates with all authors [25]. Coding was an iterative process in which generating themes was both deductive and inductive [26]. Deductive coding was based on the Technology Acceptance Model [27], Persuasive System Design Model [28], and literature on cancer-related fatigue [29]. The following main themes were identified: (1) course of cancer-related fatigue, (2) attitude toward CBM, (3) preferences with regard to the training, and (4) implementation of the training in the regular treatment course. Qualitative data analysis and research software ATLAS.ti 8.4 (Scientific Software Development GmbH) was used.

### Research Ethics

The Medical Ethical Review Committee of Medical Spectrum Twente judged that this study does not fall under the scope of the Medical Research Involving Human Subjects Act (WMO) (K18-47). The Advisory Committee on the Local Feasibility of Scientific Research ZGT agreed by providing a declaration of no objection (ZGT18-44). This study was also approved by the ethical committee of the Faculty of Behavioral, Management and Social Science, University of Twente (file number 18791). Written informed consent was obtained from all participants.

## Results

### Sample Population

This study comprised a purposive sample that consisted of 3 female patients with breast cancer with mean age of 51.33 (SD 4.71) years (a 48-year-old saleswomen from a fashion store, a 48-year-old secretary, and a 58-year-old youth doctor), 2 female patient advocates with mean age of 63 (SD 5) years, and 7 health care professionals with mean age of 50.43 (SD 7.5) years (females 6/7, 86% and males 1/7, 14%), that is, an oncologist, oncology physiotherapist, nurse practitioner, surgical oncologist, general practitioner, and 2 oncology nurses.

### Course of Cancer-Related Fatigue

In the interviews, all patients reported to suffer or have suffered from fatigue, which they experienced as a large burden in daily, social, and occupational life.

…You lose your independence as a result. Because if you're so tired, then you won't even be able to go to the supermarket to get your groceries.

Patient 1

Professionals confirmed that fatigue is very common in patients with breast cancer.

…I think that fatigue is the only complaint that affects everyone.

Nurse Practitioner

Patients described fatigue in terms of mental aspects (2 of 3 patients, eg, not being able to concentrate), emotional aspects (2 of 3 patients, eg, frustration), and physical aspects (3 of 3 patients, eg, loss of endurance). They all reported cancer-related fatigue as different from regular fatigue.

…Normally you go to bed tired and wake up refreshed, but that is not the case. Everything takes a lot of effort and energy.

Patient 1

Furthermore, professionals mentioned that fatigue often happens suddenly.

…For example, they have to walk to the counter to get a knife and then they don't know how to get there.

Surgical Oncologist

Regarding the course of fatigue, general participants indicated that fatigue is prevalent before, during, and after chemotherapy. Professionals mentioned that fatigue sometimes starts before the chemotherapy because of the diagnosis, poor sleep, and many hospital appointments. Professionals reported that severe fatigue starts during chemotherapy.

…When we give chemotherapy, I think that fatigue occurs relatively quickly, after cycles one and two and maybe three.

Nurse Practitioner

Patients (Patient 1 and Patient 2) noticed patterns of fatigue within a treatment cycle.

…The first two days were pretty good, and the third day it started to collapse. On the fourth and fifth day I felt usually very bad and tired. After that I started to feel a bit better again.

Patient 1

However, professionals mentioned that the more treatment cycles patients have had, the longer it takes for patients to recover.

…As chemotherapy continues, they become more tired and it takes longer to recover.

Oncology Nurse 2

This fact was also acknowledged by all the patients.

…I was sick during the first course of chemotherapy, but I always recovered after a cycle. However, the second course was very tough. I did not recover anymore; the fatigue was progressive.

Patient 3

Professionals reported that fatigue often persists after treatment.

…It will stick around for a very long time after completing chemotherapy.

Oncologist

…A year after treatment, it is exceptional that fatigue is not presented as the main complaint.

Surgical Oncologist

This was confirmed by the patient who had completed treatment several years ago.

…I never became my old self again after chemotherapy. [...] I had hoped that the fatigue would disappear over time, but that did not happen. So I had to adjust my life to that.

Patient 3

### Attitude Toward CBM

Health care professionals mainly reported positive opinions about the concept of CBM.

…That sounds very simple. [...] It is also a small time investment.

General Practitioner

Health care professionals indicated that they were curious about the results and that it could be fitting for patients with breast cancer.

…At a certain point, the fatigue also starts to belong to them. [...] Patients with breast cancer want to do everything they can, a good patient group in that regard.

Nurse Practitioner

Health care professionals also seemed hopeful.

…Even if it could reduce a little bit of the experienced fatigue, we have gained a lot, because it really is a big problem. Not only for the patient, but also for us as a society.

Surgical Oncologist

Furthermore, patient advocates reported that CBM could be interesting for patients who do not want to reach out for psychotherapeutic help.

…Some patients are reluctant to go to a psychologist. Maybe they can benefit in this way.

Patient Advocate 2

In general, all 3 patients reported to be open and, to some degree, positive toward the concept of CBM.

…There is no harm in trying.

Patient 1

…I think it will give me a boost.

Patient 2

They mentioned that they were curious and liked the simplicity of this concept. Two out of 3 patients reported that they would be open to receive CBM training.

…Because you want to do everything to endure the cancer treatment as good as possible.

Patient 1

One patient was very critical, reporting that she would not use the training without being informed about how it could work. She believed that fatigue was something that happened to her over which she had no control over herself, and therefore, this simple tool evoked resistance.

…As if fatigue is a choice.

Patient 3

Furthermore, she reported that it was shocking to hear that she could influence the fatigue symptoms herself, especially because she suffered from fatigue for many years after treatment. Nevertheless, she indicated that she was curious and might need some more substantiation before accepting the CBM training.

### Preferences With Regard to the Training

In the interviews, a smartphone app was mentioned as the preferred platform for the CBM training by all the participants. The training is based on repeated pairing of vitality-related words (eg, energetic) with “self” and fatigue-related words (eg, exhausted) with “others” in order to strengthen a vitality rich self-concept. On a smartphone app, one can simply swipe the words toward the correct category on the screen instead of pressing a destined key on the keyboard. The interview outcomes revealed important requirements to be taken into account in the development of the app in cocreation. All user requirements are shown in Table 2.

**Table 2:** User requirements for the training based on the interview outcomes.

User requirements	Examples of interview quotes
The app contains an introduction video that explains the rationale behind the training and explains how to perform the training.	*…I think that a video and spoken explanation will stick better in mind.* [Oncology Nurse 1] *…It would be important to know the idea behind the swiping task, otherwise it doesn’t make sense.* [Patient 1] *…Maybe I need a little more substantiation. The explanation why this would work.* [Patient 3]
One training session lasts no longer than 5 minutes (ie, no more than 100 words).	*…A training should not last longer than 5 minutes.* [Patient Advocate 2]
In the training, the categories are displayed on the top and bottom of the screen.	*…I can also imagine that you bring “energy” toward you and push “fatigue” away from you.* [Oncologist]
After completing a training session in the app, a positive message is displayed.	*…Simply a “thank you” or “well done” after you finished a session.* [Patient 2]
The app includes a progress bar that could be switched on/off.	*…That you can see how many words you have swiped.* [Patient 3]
The name and appearance of the app are positive, feminine, and simple.	*….It must be positive, but do justice to what people go through.* [Patient 3]
The app contains the option to set a daily reminder.	*…A reminder would be helpful. I notice that I quickly forget things.* [Patient 2]
The app is visually attractive and uses warm colors.	*…The more attractive it looks, the more fun it becomes to do the training.* [Patient Advocate 2] *…Use of warm colors is always good.* [Patient 2]
The app has a simple layout and is straightforward to use.	*…It should be mainly functional.* [Oncology Nurse 2] *…Keep it simple and clear.* [Oncology Physiotherapist]

Based on the requirements in Table 2, the development of the eHealth app “IVY” was started, which stands for Implicit VitalitY. The first version of the app was extensively tested and reviewed in a second iteration by 2 patients. This study continued with this version of the app, because no points of improvement were found.

### Implementation of CBM

All health care professionals would recommend the training to their patients because it is a simple and practical tool. Participants were asked how the app can be integrated into regular care so that it becomes a part of daily practice. Multiple health care professionals mentioned that the app could be included in the information session that patients attend before the start of chemotherapy in the neoadjuvant setting.

…A nurse will give them information about chemotherapy on the basis of the patient information folder [...] and fatigue is also discussed there.

Nurse Practitioner

Professionals expected the app to be helpful.

…I think that it is pretty good to give nurses a practical tool to add to the information, that might help patients on their way.

Nurse Practitioner

Patient advocates mentioned that the introduction of the app is crucial.

…Everything depends on the explanation and introduction. I think it is very important to emphasize that the training can help patients to endure that phase of therapy better, and perhaps also has some positive consequences at a later stage.

Patient Advocate 2

Nurses indicated they would be open to introduce and explain the app to patients because it connects to the information that they already provide and requires only a small time investment. Although not necessary, they mentioned that they would like to keep track of the app use once in a while.

…We can give patients some support, by just asking about it or motivating them.

Oncology Nurse 2

With regard to actually start CBM training using the app, the best option was considered to be just before the second chemotherapy cycle.

…The first time they get chemotherapy they just do not know what to expect and are often quite scared. [...] At the start of the second cycle they are usually much calmer.

Oncology Nurse 2

## Discussion

### Summary

The aim of this study was to design a CBM training to prevent chronic fatigue and improve implicit vitality in patients with breast cancer. This was a cocreation process in which patients, patient advocates, and health care professionals were involved.

### Time Point for Offering the CBM Training

The first aim was to discover the window of opportunity in the patient’s journey to offer the CBM training pre-emptively. Interviews showed that fatigue seems to increase and become more persistent as chemotherapy continues, which confirms the findings of previous research studies [30,31]. In line with a study by Spichiger et al [32], fatigue seems to be most prevalent from the third and fourth treatment cycle onwards. Our findings clearly show that the earliest suitable stage to introduce the CBM app is shortly after the start of chemotherapy but not before the first cycle. At this stage, patients have gone through the initial shock of receiving their diagnosis. They may be less apprehensive about chemotherapy after having experienced the first cycle. Additionally, for some patients, the first fatigue symptoms have already set in, motivating them to engage in a treatment. However, these findings about the preferred moment of introduction of IVY does not inform us about the point in the treatment for achieving the best effects regarding fatigue symptoms. In this regard, an effect study is needed.

### Stakeholder Perspective on CBM Training for Vitality

The second aim was to examine the attitude of the stakeholders with regard to the concept of CBM in preventing fatigue, which has not been studied before. Overall, participants showed a positive, open, and curious attitude toward CBM, with remarkably little skepticism or resistance. The health care professionals perceived the training as a simple and useful tool for providing advice to their patients. The health care professionals found CBM training to be helpful in providing patients with something they can do by themselves in this stage. Patients often feel frustrated and helpless about undergoing treatment and feel unable to do something about reducing the side effects such as fatigue in this phase. Offering a simple tool to tackle fatigue themselves seems to enable patients regain a sense of self-control, regardless of the actual effects of the training. An interesting finding was that the CBM concept evoked resistance in 1 patient. This patient attributed the fatigue to external factors only and thus was hesitant about the idea that fatigue can be influenced by oneself. It was shocking for her that such a simple preventive task could have influenced fatigue—a symptom that she suffered from for several years that had forced her to adjust her life completely. This might be interpreted as the first indication for the beneficial effects of preventive use of CBM training for vitality in order to buffer the development of an unbeneficial cognitive bias for fatigue. However, it should be taken into account that the training can possibly lead to self-blaming in patients [19]. On the one hand, self-blaming might lead to negative affect. On the other hand, blaming oneself might enhance perceptions of control [33]. Nevertheless, it seems relevant to inform patients about the automaticity and lack of awareness that characterize cognitive biases to avoid harmful interpretations by patients. Such educational content might easily be included in the app.

The third aim of this study was to explore the preferences with regard to the CBM training. A smartphone app was the preferred platform for the training. Interestingly, participants spontaneously suggested a vertical positioning of the CBM categories, with the “Self-Vitality” combination at the bottom and the “Other-Fatigue” stimuli on top, thereby allowing a more intuitive swipe movement toward or away from their body accordingly. Although the self-as-fatigued identity CBM was originally based on the implicit association test paradigm, this adds an approach-avoidance mechanism that originates from a different CBM paradigm [34]. A classical implicit association test is considered to represent evaluative associations with the target—in this case, the concepts of fatigue and vitality, stored in memory after repeated previous experiences [35]—and is conceptually most closely related to an attitude as a cognitive concept [36]. The approach-avoidance task is thought to capture cognitive embodiment, which is conceptually closer to motoric impulses [37,38]. By combining both the evaluative conditioning from the implicit association test paradigm and the motoric tendencies from the approach-avoidance task paradigm, multiple mechanisms may be targeted, which could potentially enhance the effectiveness of the intervention.

Additional requirements that emerged from the interviews can be explained in the light of the persuasive system design model [28], which defines several persuasive design principles. From the category of primary task support, there seemed to be a need for self-monitoring (progress bar) and reduction (simple layout). From the category dialogue support, there seemed to be a need for liking (visually attractive), praise (positive messages), and a reminder. From the category credibility support, there seemed to be the need for expertise by offering a good explanation for the rationale behind the training. No findings related to the category of social support emerged in line with the individual nature of the training task. Overall, the simplicity of the training appeared to be the major strength. This implies that adding persuasive elements such as gaming features may not improve usability. In line with Hassenzahl’s hedonic/pragmatic model of user experience, this suggests that patients expect health technology to be pragmatic rather than hedonic in nature [39]. Based on all the requirements, the eHealth app IVY was created. The link to the app is shown in Multimedia Appendix 1. With regard to the implementation, it is recommended that IVY be aligned carefully with clinical treatment. Furthermore, it is recommended that health care professionals are involved to foster successful implementation. Nurses seemed willing to provide oral explanation about the app and mentioned that it could be included in patient education materials.

### Strengths of This Study

A strength of this study is the user-centered design approach. The design of IVY was a cocreation process, involving a broad range of stakeholders, including patient advocates at an early stage in order to optimize successful implementation at a later stage. This study is innovative in multiple ways. First, this study is the first to develop a CBM intervention for fatigue for patients with cancer undergoing treatments. Second, this is the first study that focuses on developing a preventive CBM intervention instead of working on the existing reactive CBM interventions. Currently available CBM evidence is limited to attempts to correct already established maladaptive response tendencies. However, assuming that the concepts of fatigue and vitality are to some degree ends of a single dimension, it seems plausible that training patients toward the positive end would create a buffer against the development of adverse conditioning later on during the disease. Even when both concepts would be rather separate dimensions, strengthening vitality bias may still help attenuating adverse effects of fatigue bias, as has been shown convincingly by the two-continua approach in the positive psychology field: building positive mental health has shown to reduce the incidence of psychopathology or to protect against its impact on the quality of life [40,41]. Lastly, by creating a mobile CBM intervention, this study is also innovative within the CBM research field.

### Limitations of This Study

A limitation of this study is the small sample size, especially of the patient group (n=3). However, the patient advocates are patients with breast cancer too. They were invited as representatives and stakeholders of BVN. The mean age of the patients seemed representative, considering the regional hospital population and in relation to the Dutch Institute for Clinical Auditing registry. Therefore, our study includes a broader patient perspective than that based on the input of 3 patients participating on personal title. This sample is expected to be sufficient for the goal of this study, which is an intervention design. Furthermore, health care professionals were able to position themselves in the place of patients and as such, may also represent the patient perspective in certain way. Besides, the reach of saturation in the answers is a valid indicator of sufficient sample size [42]. Representativeness with regard to age is a limitation of this study since patients with breast cancer younger than 40 years or older than 70 years did not take part in the cocreation of IVY.

The second limitation of this study is that the interviews did not provide as much information about design and content as was expected. Participants were overall quickly satisfied and not very critical. A possible explanation for this is that participants had no prior knowledge about persuasive elements and simply had no idea about all the options. Future research studies should give more examples about persuasive elements to inspire patients to come up with their own ideas and preferences.

The last limitation of this study is that the verbal stimuli that are used in the app were not pretested among the participants of this study. However, the stimuli were collected, pretested, and validated in previous studies [43-45] in healthy individuals. The verbal stimuli were retrieved from both items in validated questionnaires for measuring the constructs of fatigue and vitality and from interviews. In this study, no negative responses to the stimuli were observed, confirming the face validity of these materials. However, in further cocreation research, it would be recommended to evaluate these stimuli among this patient population as well.

### Clinical Implications

Patients suffering from fatigue are often reluctant to receive time-consuming and energy-consuming interventions such as cognitive behavioral therapy, especially during their treatment. Moreover, these therapies are not widely available owing to limited clinical capacities or insurance coverage. This simple CBM swipe training requires low levels of mental energy and literacy. Furthermore, patients can complete the training anywhere and anytime on their own smartphone, which makes it very time-saving and cost-effective. IVY is expected to be a low-threshold intervention that is suitable for many people. When the implicit vitality training shows to be effective, it could also be applied to other cancer types, populations, or chronic diseases accompanied with serious fatigue symptoms. The unique characteristic of IVY is that it can be used as a preventive intervention in the early stage of cancer treatment unlike most existing fatigue programs that target already manifested fatigue. The aim of this psychological conditioning task is that the self-concept becomes more focused on vitality, which aims to create a buffer to protect against the fatigue symptoms that will occur during treatment and recovery. This pro-vitality self-image will then contribute positively to recovery, promoting behavior, and to quality of life. Taking into account that fatigue is one of the most prevalent symptoms in patients with cancer, even small progress could be clinically relevant.

## Appendix

Multimedia Appendix 1 Supplementary material.

## Abbreviations

BVN: Dutch breast cancer patient association
CBM: cognitive bias modification
IVY: Implicit VitalitY
ZGT: Ziekenhuis Groep Twente
