# Supplementary material for: Cognitive Bias Modification Training to Improve Implicit Vitality in Patients With Breast Cancer: App Design Using a Cocreation Approach
Source: JMIR Form Res. 2021 Mar 10;5(3):e18325. doi: 10.2196/18325 (PMC7991988; doi:10.2196/18325)
Supplement: Multimedia Appendix 1 [file formative_v5i3e18325_app1.docx]

**Supplementary material:**

**Literal translation of description of study and CBM from original Dutch material**

***Aim of this study:***

In the Netherlands, more and more women with breast cancer are receiving intensive treatment for this. After such treatment, they often suffer from severe fatigue complaints. There are a number of tools that teach these women to monitor consciously their behavior and its consequences. However, despite these interventions, many women cannot function as before. Often different self-image, associated with fatigue, has been developed. In this research we will design an application, which will consist of a simple repeated training, with which we want to prevent that the self-image of breast cancer patients focuses too much on fatigue. Of course we want the application to connect well that match the patients’preferences since they are the group that will use the app in the future.

For our project we would like to ask you a number of questions, for example about how fatigue plays a role in your life. We would also like to hear your opinion and wishes about the application we are going to develop. We think you can give us very valuable information.

***Explanation CBM:***

Then I will now briefly explain to you about the application we intend to develop.

We humans have both conscious and unconscious processes (show: very famous picture with mountain above and below the water) that determine our behavior. Conscious processes are related to we want, think or feel consciously. Unconscious processes are the more automatic processes, such as habits. As you can see, much of our behavior is driven by unconscious processes.

Many existing interventions focus on the conscious processes. Because unconscious processes determine such a large part of the behavior, we will focus our application on these processes. We actually want to relearn an automatic response with this application.

Example: Biscuit with coffee: if you always take a biscuit with coffee, the two automatically go together. When you walk to the coffee machine, you automatically (and unnoticed) feel the urge for a cookie (and vice versa). This is an association created by reward (learning effect).

These processes also work with negative associations, such as in the experience of fatigue.

This is a learned reaction, so you can change it again by learning. This is what we want to do in this application, concentrating on the self-image of patients that automatically (and often too strongly) focuses on fatigue. We want to relearn this response, so that patients' self-image becomes less focused on fatigue and more focused on vitality.

An example of an app that has been developed before and which also focuses on the automatic and unconscious processes is “Breindebaas”: an app for people with an alcohol problem / addiction. These people often have an automatic approach to alcohol. In this app, this automatic reaction is relearned by allowing users to swipe pictures of alcohol away and pictures of fresh / water towards them. This app has already shown positive results in a pilot study; people have actually started drinking less.

In the app that we are going to develop, we will use the same technique, but participants will not have to swipe images, but link certain words to each other. They should associate words like "self" and "I" with words like "fit," and words like "others" with words like "tired." The training consists of a lot of repetition and the intention is that patients do this daily for two weeks (10 min. Per day).

The idea behind the app is that breast cancer patients often develop a distorted self-image that is too focused on fatigue. With this app we try to change this bias more towards vitality. We also expect an indirect effect, namely that patients will also exhibit behavior which in time will also contribute to strengthening vitality.

The app is therefore not aimed at the conscious processes, like most existing interventions

but on the unconscious and automatic processes.

Example of how this looks on the computer:

**

**Supplementary material: Link to the IVY application**

[*https://play.google.com/store/apps/details?id=com.evolution36.ivy&hl=nl*](https://play.google.com/store/apps/details?id=com.evolution36.ivy&hl=nl)

**Supplementary material to table 1:**

**Literal translation of interview questions from original Dutch material**

| Age, occupation, diagnosis & treatment | What is your age?; Can you briefly indicate what you do (or have done) in daily life?; Can I ask when you have been diagnosed with breast cancer?; What treatment did you receive for breast cancer? |
| --- | --- |
| Impact, course of fatigue | What does "fatigue" mean to you?; To what extent is there a difference between the fatigue related to breast cancer and the fatigue that you knew before?; To what extent have you noticed that fatigue played a role during your illness?; When did these fatigue complaints start? And what was the course of this?; Are these complaints still present?; What impact does this fatigue have (had) on your life?; Can you give examples of this?  Do you have an idea about why fatigue does occur in some patients and not in others?; To what extent is fatigue a topic that is discussed in the contact between the patient and the healthcare professionals? ; What do you think about this? How did you experience this? Suggestions / points for improvement?; Do you know of interventions that focus on fatigue in patients?; Do you have an idea of ​​what such an intervention aimed at fatigue should look like? Suppose you can come up with an intervention yourself, what is important, etc. |
| Attitude and experience supporting health by technology and opinion CBM training | When we talk about technology in healthcare, it is very broad, you may think first of all about scanners and devices. But technology also includes the use of online programs and apps to support regular treatments. That's what this interview is about. What do you think of this?; Do you already have experience with the use of technology (eg apps)?; If so, what is your experience? Can you describe this? |
| Preferred platform, content, lay-out and explanation of the training | General impression of low-fi prototype figure:  What do you think about this? Can you explain why?; To what extent do you think patients will benefit from this?; How do you think patients respond to this? Do you think they like this?  Explanation of training:  To what extent do you think there should be an explanation for the patient about the practical implementation of the app? And in what way? (e.g. text, pictures, video); To what extent do you think there should be an explanation for the patient about the idea behind the app? (rationale) And in what way? (e.g. text, pictures, video)  Content app:  To what extent do you think that repeated training influences motivation?; To what extent do you think repetitive training is a problem for patients?  One possibility is to add motivational elements to the app.  Suppose there is a continuum :Purely functional app - Midfield - Game  If you keep this in your mind, to what extent do you think motivation elements should be added to the app? What would patients find attractive? And what would it take?  Do you have an idea about which elements? And which ones are not?  [think of reminders, feedback, virtual coach / avatar etc.]  The idea is to have patients train daily in the app for a period of 2 weeks, about 10 minutes a day.  What do you think of the length of the training?; What do you think of the number of times patients have to train? (14 days); Do you think this is feasible for patients? Or would you rather see this differently? |
| Integration of training in regular care and the best moment of offering it to patients | Implementation:  What do you think of the reliability and credibility of the app?; What would make patients experience the app as more reliable and credible?; Would you use the app if it were offered in your situation? To what extent do you think your patients would like to use this app?; What do you think should change?; Suppose it works: what do you think we should do to really integrate the app into the care process?; The intention is to use the app preventively. This means that patients will start using the app before the start of the first chemo (immuno) treatment. Do you have an idea at what time it is best to offer the app to the patient before the start of the first chemo (immuno) treatment? |
